# Extracellular vesicles and its advances in female reproduction

**DOI:** 10.21451/1984-3143-AR2018-00101

**Published:** 2020-05-22

**Authors:** Ana Clara Faquineli Cavalcante Mendes de Ávila, Gabriella Mamede Andrade, Alessandra Bridi, Lindsay Unno Gimenes, Flávio Vieira Meirelles, Felipe Perecin, Juliano Coelho da Silveira

**Affiliations:** 1 Department of Veterinary Medicine, Faculty of Animal Sciences and Food Engineering, University of São Paulo, Pirassununga, SP, Brazil.; 2 Department of Preventive Veterinary Medicine and Animal Reproduction, School of Agricultural and Veterinarian Sciences, São Paulo State University (Unesp), Jaboticabal, SP, Brazil.

**Keywords:** extracellular vesicles, intercellular communication, female reproduction

## Abstract

Intercellular communication is an essential mechanism for development and maintenance of multicellular organisms. Extracellular vesicles (EVs) were recently described as new players in the intercellular communication. EVs are double-membrane vesicles secreted by cells and are classified according to their biosynthesis, protein markers and morphology. These extracellular vesicles contain bioactive materials such as miRNA, mRNA, protein and lipids. These characteristics permit their involvement in different biological processes. Reproductive physiology is complex and involves constant communication between cells. Different laboratories have described the presence of EVs secreted by ovarian follicular cells, oviductal cells, *in vitro* produced embryos and by the endometrium, suggesting that EVs are involved in the development of gametes and embryos, in animals and humans. Therefore, is important to understand physiological mechanisms and contributions of EVs in female reproduction in order to develop new tools to improve *in vivo* reproductive events and assisted reproductive techniques (ARTs). This review will provide the current knowledge related to EVs in female reproductive tissues and their role in ARTs.

## Introduction

Intercellular communication is an important reproductive physiology event. Cells that form reproductive tissues and structures are in constant interaction influenced by endocrine, paracrine and autocrine signaling (Albertini and Rider, 1994; Kretser *et al*., 2002). Besides the traditional forms of intercellular communication several studies have described extracellular vesicles (EVs) mediating the crosstalk between cells within or among tissues (da Silveira *et al*., 2012; Al-dossary *et al*., 2013; Ruiz-Gonzalez *et al*., 2015). Extracellular vesicles are nanoparticles secreted by different cell types (György *et al*., 2011; Raposo and Stoorvogel, 2013) and contains bioactive molecules such as mRNAs, microRNAs and proteins, which participate in the intercellular communication (Valadi *et al*., 2007; Simpson *et al*., 2008). 

Extracellular vesicles secretion has been described in diverse reproductive cells as follicular cells (da Silveira *et al*., 2012), oviductal cells (Al-dossary *et al*., 2013), embryos produced *in vitro* (Mellisho *et al*., 2017) and endometrium cells (Ng *et al*., 2013). Moreover, many reproductive physiological functions are associated with EVs. These functions include ovarian follicle development, oocyte maturation and fertilization, early embryo development and endometrial-conceptus crosstalk (da Silveira *et al*., 2012; Al-dossary *et al*., 2013; Ruiz-Gonzalez *et al*., 2015; Hung *et al*., 2015; Lopera-vásquez *et al*., 2016). Moreover, EVs has also emerged as a potential *in vitro*-tool to improve assisted reproductive techniques (ARTs).

Assisted reproductive techniques are frequently used in animals and humans (Sirard, 2018). The principals of its use are to improve reproductive efficiency and as an alternative to infertility (Bousquet *et al*., 1999). However, exposure of gametes and early embryos to the *in vitro* environment influences embryo quality, RNA expression and ultrastructure patterns (Rizos *et al*., 2002a,b). These changes could cause pregnancy losses and can affect the health of generated individuals (Urrego *et al*., 2014; Bouillon *et al*., 2016). Therefore, is important to understand the physiological events in order to improve ARTs by mimicking *in vitro* the physiological conditions.

In summary, ARTs are important reproductive techniques; however, the *in vitro* environment can negatively affect gametes, embryo development and pregnancy establishment. One of the reasons is related to the lack of physiological mechanisms such as intercellular communication. Extracellular vesicles are carriers of molecules and were demonstrated to affect follicle development, oocyte maturation, embryo production and endometrial-conceptus communication. Then, this review will demonstrate the main studies describing EVs related to female reproductive tract and their impact in ARTs.

## Extracellular vesicles characterization

Extracellular vesicles (EVs) are membrane vesicles formed by a lipid bilayer that are secreted by cells (Théry *et al*., 2009). They are found in body fluids and include exosomes, microvesicles and apoptotic bodies (György *et al*., 2011). Extracellular vesicles types can be differentiated by size, biosynthesis and contents. These membrane vesicles are released in the extracellular environment in response to specifics stimulus in physiological or pathological situations (Cocucci *et al*., 2009; El Andaloussi *et al*., 2013; Tannetta *et al*., 2014). The collection and study of only one subtype of EVs is still a challenge due to the lack of specific markers and protocols for isolation (Gould and Raposo, 2013; Lötvall *et al*., 2014). Thus, it is recommended to use the term extracellular vesicles. 

Exosomes are small EVs between 30-150 nm in diameter (György *et al*., 2011). Its biosynthesis involves the formation of multivesicular bodies (MVBs) from a first plasma membrane endocytosis, forming the early endosome. Intraluminal vesicles (ILV) are formed into MVB and are released in the extracellular environment after the fusion of MVB with the plasma membrane (Théry *et al*., 2002; 2009). This mechanism was described firstly during *in vitro* maturation of reticulocytes (Harding *et al*., 1983; Pan *et al*., 1985). Differently, microvesicles are large EVs between 100-1000 nm in diameter formed from plasma membrane blebbing in response to stimulus or stress (Cocucci *et al*., 2009; Tannetta *et al*., 2014). Apoptotic bodies are heterogeneous vesicles, from 1-5µm originated from apoptotic processes and contain organelles and nuclear fragments (Pavani *et al*., 2017). Curiously, new subpopulations of nanoparticles were described in a recent study (Zhang *et al*., 2018). They were termed small exosomes vesicles (60-80 nm), large exosomes vesicles (90-120nm) and exomeres (~35 nm) (Zhang *et al*., 2018). The study suggested distinct biological function for each subsets of nanoparticles, since its different biodistribution patterns (Zhang *et al*., 2018).

Despite the divergences in nomenclature between the subtypes of EVs, exosomes and microvesicles have important roles in biological processes and have been largely studied. Extracellular vesicles, such as exosomes and microvesicles, have bioactive material such as mRNA and microRNAs (Valadi *et al*., 2007), proteins (Simpson *et al*., 2008) and lipids (Subra *et al*., 2007). These components suggest their cell modulating functions. Additionally, the mechanism of communication between EVs and target cell include, briefly: 1) Interaction between membrane proteins activating intracellular signaling within target cells; 2) Cleavage of membrane exosomal proteins near to target cell receptors; 3) molecular transfer of EVs contents by fusion with target cell and 4) EVs phagocytosis by recipient cells (Mathivanan *et al*., 2010; El Andaloussi *et al*., 2013). 

Extracellular vesicles can be obtained from extracellular fluids derived from culture media or body fluids (Lötvall *et al*., 2014). Several methods for isolation are in constant discussion. The ideal method can vary according to the question to be answered, application, purity and desired concentration (Witwer *et al*., 2013; Lötvall *et al*., 2014). The appropriated methods for EVs isolation and analysis are standardized by the International Society for Extracellular Vesicles (ISEV). Usually, the techniques to isolate EVs are ultracentrifugation, filtration, polymer precipitation, immunoaffinity and microfluidic techniques (Witwer *et al*., 2013). The analysis of isolated products can be quantitative and qualitative. It can be realized by electronic and atomic force microscopy, optical particle tracking, flow cytometry and western blotting; to evaluate morphology, size, concentration, purity and protein content, respectively (Witwer *et al*., 2013). 

## Small extracellular vesicles in female reproduction

Since the observations of small EVs in intercellular communication, many studies have shown the influence of these vesicles in reproductive processes. In this context, small EVs were described in follicular fluid (da Silveira *et al*., 2012), oviductal fluid (Al-dossary *et al*., 2013), secreted by embryos in culture media (Kropp *et al*., 2014; Kropp and Khatib, 2015a; b) and in endometrium flushing (Ng *et al*., 2013). Additionally, the knowledge of molecular mechanisms implicating EVs is favoring the development of new technologies involving biological roles, diagnostic and therapeutic potential (Saadeldin *et al*., 2015). 

### Extracellular vesicles in ovarian follicle environment

**Figure 1:**
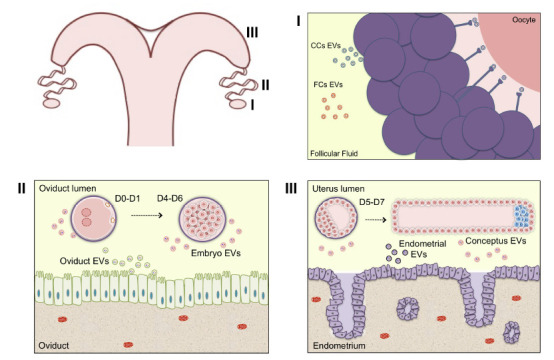
Extracellular vesicles in female reproduction. Schematic representation of extracellular vesicles present within the ovarian follicular microenvironment, as well as during the embryonic development within the oviduct and uterus. (I) Extracellular vesicles in the follicular fluid can carry and transfer bioactive molecules such as proteins, RNAs, miRNAs, lipids and metabolites contributing to oocyte maturation process, (II) to fertilization and embryonic early development in the oviductal microenvironment (III) as well as during the maternal recognition within the uterine microenvironment. Legend: EVs - extracellular vesicles; CCs - cumulus cells; FCs - follicular cells; D0 - fertilization day.)

The ovarian follicle is an important structure within the ovary. This microenvironment is composed by theca cells, granulosa cells, cumulus cells and the oocyte (reviewed by Knight and Glister, 2006). During the folliculogenesis, antral follicles are characterized by the presence of follicular fluid (reviewed by Edson *et al*., 2009). The follicle microenvironment is regulated by endocrine, paracrine and autocrine factors during its development (Albertini and Rider, 1994; Matsuda *et al*., 2012). Therefore, the intercellular communication within this microenvironment is essential for oocyte and follicle development. Additionally, follicular cells can secrete EVs, found in follicular fluid, which transmit information between cells (Andrade *et al*., 2017; Fig. 1-I). 

Small EVs were firstly described in follicular fluid of mares (da Silveira *et al*., 2012). In this study, it was confirmed the small EVs uptake by granulosa cells *in vivo* and *in vitro*. In addition, different miRNAs were found in small EVs of young and old mares (da Silveira *et al*., 2012). Interestingly, different miRNAs contents were also observed in small EVs from follicular fluid of young and older women (Diez-Fraile *et al*., 2014). Sohel *et al*. (2013) described the proportion of miRNAs contents within different follicular fluid fractions. This group demonstrated for the first time that the majority of miRNAs from follicular fluid were in the exosomes fraction (Sohel *et al*., 2013), which emphasizes the importance of studying EVs miRNA contents. Furthermore, this study showed that exosome uptake by follicular cells was associated with an increase in miRNAs levels in these cells (Sohel *et al*., 2013). 

Additionally, the communication mechanisms and effects of small EVs from follicular fluid, granulosa cells and COCs are under investigation. Exosomes from follicular fluid were involved in regulate TGF-beta (transforming growth factor beta) signaling pathway, an important pathway related to follicular development, in granulosa cells (da Silveira *et al*., 2014). This study showed that exosomes regulate members of TGF-beta pathways such as ACVR1 (activin A receptor type 1) and ID2 (inhibitor of DNA binding 2) in granulosa cells *in vitro* by transferring mRNA, protein and miRNAs (da Silveira *et al*., 2014). Recent studies demonstrated that EVs from bovine follicular fluid from small follicles (3-5mm in diameter) and large follicles (>9mm in diameter) induce cumulus expansion during *in vitro* maturation (Hung *et al*., 2015). Importantly, this study demonstrated a better effect by EVs isolated from small follicles. Additionally, EVs from small follicles supplemented during COCs *in vitro* maturation induced changes in embryo transcripts levels, increase in blastocyst rates, as well as changes in global levels of DNA methylation and hydroxymethylation (da Silveira *et al*., 2017). 

Other studies demonstrated that EVs characterization change according to follicle dimension (Navakanitworakul *et al*., 2016; Hung *et al*., 2017). Extracellular vesicles concentration and its miRNA contents are modified between small, medium and large bovine follicles (Navakanitworakul *et al*., 2016). Moreover, this study suggests changes in biogenesis or in EVs uptake during follicle development. This information was confirmed since the uptake of EVs from small follicles by granulosa cells was preferential, comparing to medium and large follicles EVs (Hung *et al*., 2017). The differences found between EVs from small and large follicles are related to increased granulosa cell proliferation after EVs supplementation from small antral follicles comparing to large follicles (Hung *et al*., 2017). 

In summary, it is clear that EVs from follicular fluid have important contents related to biological processes during follicle and oocyte development. As an example, it is possible to detect effects from these EVs in cumulus and granulosa cells (Hung *et al*., 2015, 2017). These studies increased our understanding regarding reproductive biology processes as well as the possibility of using these EVs to improve oocyte *in vitro* maturation in many species. However, the follicle environment undergoes hormonal and developmental modifications that can change EVs biogenesis and contents, thus is important to consider ovarian follicle physiology in order to improve the use of EVs in the assisted reproductive techniques.

### Extracellular vesicles in the oviduct environment

The oviduct is an important part of the female reproductive organ located between ovary and uterus (Hunter, 2012). After ovulation, the oocyte goes to the oviduct, where it undergoes fertilization and early embryo development in mammals (Spencer *et al*., 2007). The mammalian oviduct epithelium is composed by ciliated and secretory cells that are involved in these processes (Eriksen *et al*., 1994). These cells participate in secretion of oviductal fluid that has important roles in oocyte competence and embryo development (Leese *et al*., 2008; Avilés *et al*., 2010). Extracellular vesicles are one of the components of the oviductal fluid that favor oocyte and embryo quality (Lopera-vásquez *et al*., 2016; Fig. 1-II).

The presence of EVs in the oviductal fluid was described for the first time in murine and it was called “oviductosomes” (Al-dossary *et al*., 2013). Al-dossary *et al*. (2013) showed that murine oviductosomes contain a membrane protein called Plasma Membrane Ca2+ - ATPase 4 (PMCA4) that play roles in sperm capacitation, and subsequently in fertilization. Additionally, this study demonstrated *in vitro* the uptake of exosomal PMCA4 by sperm cells, suggesting an important role for these vesicles during fertilization.

In the beginnings of IVF different laboratories used oviductal cells in co-culture with embryos to mimic the beneficial effects of *in vivo* system (Eyestone and First, 1989). However, these techniques presented some disadvantages such as undefined culture conditions, embryo and somatic cells competition for nutrients as well as risk of diseases transmission (Orsi and Reischl, 2007). Thus, conditioned medium and EVs could serve as alternatives to co-culture components (Maillo *et al*., 2016). Moreover, the study of the EVs contents could lead to the use of specific donor cells or to the development of synthetic or semi synthetic vesicles to transport components of interest in order to mimic the maternal environment. 

Proteomic profile of EVs secreted by bovine oviduct epithelial cells (BOEC) *in vivo* or *in vitro* presents important differences (Almiñana *et al*., 2017). *In vivo* collected EVs presented proteins related with fertilization and early pregnancy development, like oviductal glycoprotein (OVGP), heat shock protein A8 (HSPA8) and myosin 9 (MYH9). However, the BOECs submitted to *in vitro* system did not present OVGP (Almiñana *et al*., 2017), an important protein associated with zona pellucida maturation (Avilés *et al*., 2010). Therefore, these studies suggest that the *in vitro* system can affect oviductal EVs contents. 

Extracellular vesicles supplementation to embryo culture media demonstrated functional effects in early embryo development (Lopera-vásquez *et al*., 2016; Almiñana *et al*., 2017). Additionally, it was demonstrated that *in vitro* produced embryos can internalize oviductal EVs (Almiñana *et al*., 2017; Pavani *et al*., 2017). Although, frozen collected oviductal EVs supplemented to *in vitro* culture media, improves *in vitro* blastocyst yield and quality in comparison with fresh oviductal EVs and negative control (without EVs; Almiñana *et al*., 2017). Similarly, EVs from BOEC were able to reproduce the beneficial effects of oviduct cells conditioned medium, suggesting its use as an alternative supplementation for embryo development culture media (Lopera-vásquez *et al*., 2016). Embryos treated with EVs from BOEC culture media presented greater number of total cells and better survival rate after vitrification in comparison with embryos cultured without EVs (Lopera-vásquez *et al*., 2016). Additionally, this study demonstrated a greatest relative expression of *PAG1* (Phosphoprotein Membrane Anchor with Glycosphingolipid Microdomains 1), an implantation related gene, in embryos supplemented with EVs. Specifically, oviductal fluid derived EVs from isthmus resulted in greatest bovine embryo survival rate after vitrification comparing with EVs from ampulla or in absence of EVs (Lopera-Vasquez *et al*., 2017). The improvement in embryo quality can be associated to the level of *AQP3* (Aquaporin 3), a water channel, that was upregulated in embryos supplemented with EVs from isthmus comparing with embryos supplemented with FCS only (Lopera-Vasquez *et al*., 2017).

In conclusion, oviductal derived EVs have a role during embryo early development. This communication can favor fertilization and embryo quality. Then, EVs from oviduct are possible tools to improve *in vitro* culture system. However, it is important to consider the physiological modifications in EVs contents according to oviduct origin *in vivo* or *in vitro* as well as the region of oviduct used to obtain these EVs. These concerns are important to understand the role of these EVs during biological processes such as fertilization and early embryo development aiming to use these EVs properly in different ARTs.

### Extracellular vesicles in embryo in vitro production

Embryos produced *in vitro* are exposed to different conditions in comparison with the *in vivo* oviductal and uterine environment. These *in vitro* conditions are responsible for many embryonic alterations, including morphological, metabolic, developmental and molecular changes (Plante and King, 1994; Holm and Callesen, 1998; Lonergan *et al*., 2003). Based on that, is important to investigate *in vitro* embryo derived EVs in order to understand the consequences of the *in vitro* environment. The first evidence of embryo derived EVs was demonstrated by the co-culture of porcine parthenogenetic (PA) and somatic cell nuclear transference (SCNT) embryos (Saadeldin *et al*., 2014). Pluripotency transcripts such as octamer-binding transcription factor (*OCT4*), sex determining region Y-box 2 (*SOX2)*, avian myelocytomatosis viral oncogene homolog (*C-MYC)* and homeobox transcription factor nanog (*NANOG*) were present in exosomes secreted by porcine PA embryos, suggesting that co-culture of embryos could create a microenvironment, thus improving SCNT embryo development (Saadeldin *et al*., 2014). 

Moreover, SCNT embryos also secrete exosomes in the medium during *in vitro* culture. Supplementation with exosomes secreted by SCNT embryos in the culture medium of SCNT embryos increases blastocyst rate, total cell numbers, ratio of ICM/TE as well as transcripts levels of *OCT-4* in comparison to SCNT embryos without supplementation (Qu *et al*., 2017). These findings indicated that exosomes present in the culture medium are essential for embryo development and changes caused during embryo development by culture medium replacement may be repaired by exosome supplementation (Qu *et al*., 2017). Thus, *in vitro* embryo derived EVs may modulate *in vitro* conditions creating a microenvironment that favors embryonic development. Additionally, embryo derived EVs could serve as non-invasive marker correlated with embryo quality. 

### Extracellular vesicles in endometrial-conceptus cross-talk

Since early pregnancy stages, the establishment of cross communication between embryo/conceptus and the maternal organism is necessary. The endometrium is formed by epithelial and glandular cells capable to secrete nutrients and growth factors essential for early embryonic development (Bazer *et al*., 2011). Additionally, in ruminants, conceptus secreted interferon-tau (IFNT) is a protein responsible for signaling conceptus presence inside the maternal uterus during maternal recognition of pregnancy (Farin *et al*., 1990). 

Extracellular vesicles are part of this cross communication important for maternal recognition of pregnancy (Tannetta *et al*., 2014). During pre-implantation period conceptus and maternal endometrium can secrete EVs (Saadeldin *et al*., 2015; Fig. 1- III). In order to test that, on day 14 of pregnancy, uterine flush (UF) derived EVs were labeled with PKH67 and infused back in the pregnant uterus. Upon collection of tissues, conceptus trophoblast and uterine epithelia contained labeled EVs, suggesting the role of EVs in the cross communication between those cells at the onset of pregnancy (Burns *et al*., 2016). Similarly, uterine epithelial cells can secrete EVs containing ovine endogenous jaagsiekte retroviruses (*enJSRV*) mRNA (Ruiz-González *et al*., 2015). Free and exosomal enJSRV act via toll-like receptors (*TLR*) on the conceptus trophectoderm cells (oTr1) to induce interferon-tau (IFNT) secretion (Ruiz-González *et al*., 2015). Trophoblast CT-1 cells treated with exosomes from uterine flushing of pregnant cows on Days 17, 20 and 22 did not change *IFNT* and caudal type homeobox 2 (*CDX2*) transcripts levels, suggesting an influence of the pregnancy period on the components of the EVs (Kusama *et al*., 2018). 

Moreover, ovine conceptus trophectoderm derived exosomes, with 15 and 17 days of pregnancy, have IFNT, macrophage-capping protein (CAPG) as well as aldo-keto reductase family 1, member B1 protein (AKR1B1) (Nakamura *et al*., 2016). Similarly, exosomes secreted by ovine and bovine conceptus containing IFNT were able to increase interferon-stimulated genes (ISGs) mRNA expression in primary uterine endometrial epithelial cells (EECs) (Nakamura *et al*., 2016; Kusama *et al*., 2018). Endometrial epithelial cells treated with exosomes at pre-implantation period (P17 UFs) increased the expression of apoptosis - related genes such as BCL2 associated X, apoptosis regulator (*BAX)*, caspase 3 (*CASP3)*, tumor necrosis factor (*TNFA*) and tumor protein P53 (*TP53*) transcripts (Kusama *et al*., 2018). Besides that, exosomes at post-implantation (P20 and P22 UFs) stimulate adhesion molecules such as vascular cell adhesion molecule 1 (*VCAM1*) mRNA (Kusama *et al*., 2018). These results demonstrate that changes in the uterine environment mediated by exosomes are required for attachment and development of the conceptus.

Progesterone and estradiol hormone can influence the secretion of exosomes by endometrial cells in human (Greening *et al*., 2016). Progesterone is needed for elongation and survival of ovine conceptus (Spencer and Bazer, 2002). Thus, EVs number increases from Day 10 to 14 post-estrus in the ovine uterine lumen (Burns *et al*., 2018). Moreover, progesterone treatment increased the EVs number in the uterine lumen in comparison with a P4 receptor antagonist group (Burns *et al*., 2018). MiRNAs upregulated by progesterone in ovine uterine EVs are predicted to modulate pathways such as PI3K/AKT, bone morphogenetic proteins (*BMP*) and post-transcriptional silencing by small RNAs (Burns *et al*., 2018). These findings indicate a new way by which P4 may modulate endometrial function controlling conceptus growth during pregnancy establishment.

On Day 20 of pregnancy, EVs were identified in the porcine endometrium and chorioallantoic membrane (Bidarimath *et al*., 2017). Porcine trophectoderm derived EVs induce aortic endothelial cells proliferation, which may be stimulating angiogenesis (Bidarimath *et al*., 2017). Furthermore, porcine trophectoderm and aortic endothelial cells EVs have miRNAs predicted to modulate angiogenesis and placental development pathways, suggesting that these EVs may play an important role in the communication between conceptus and maternal endometrium influencing the establishment of pregnancy (Bidarimath *et al*., 2017).

Therefore, factors such as hormones and the day of pregnancy can change EVs secretion and contents in the uterine environment. Based on that, is important to understand the mechanisms modulating uterine EVs, which can impact protein, mRNA and miRNAs contents involved in important biological processes during early pregnancy in the different domestic species. Finally, uterine EVs may play important role in endometrial-conceptus cross-talk in the period of maternal recognition of pregnancy and might be involved in pregnancy success.

## Perspectives

Since the discoveries of EVs biological functions, many studies have been conducted exploring their biosynthesis, contents, interaction with recipient cells as well as methods for isolation and characterization. Thus, is important to clarify the involvement of EVs in physiology and pathological processes. Additionally, EVs have potential as new biomarkers and therapeutics. Different studies are exploring the inhibition of EVs formation, release and uptake by recipient cells, as well as their potential in therapeutic (El Andaloussi *et al*., 2013). However, we still have challenges on these subjects. Despite the challenges, the development of synthetic and semi-synthetic vesicles might be the mechanism to use these EVs *in vivo* and *in vitro*. Extracellular vesicles are considered a new biological strategy to reach cells and deliver nucleic acids with increased specificity (Nguyen and Szoka, 2012). Despite the good perspectives for the clinical application of EVs, the success depends on EVs time of processing, the small volumes of fluids, the knowledge regarding EVs trafficking and functions on the *in vivo* environment (Hood and Wickline, 2012). 

In the reproduction scenario, synthetic or semi-synthetic EVs are yet to be developed. However, since their description in reproductive fluids, many researches have demonstrated cross communication involving EVs as well as the contents such as miRNAs, mRNAs and proteins in different reproductive biofluids. Therefore, is important to increase our knowledge regarding the EVs biology in reproductive tissues in order to generate new approaches to improve fertility. The goal will be to create a better environment to produce embryos *in vitro* and consequently generate healthier pregnancies in animals and humans. 
